# Management of acid and alkaline phosphatase, dehydrogenase activities by sugarcane industry waste under lead contamination- A case study of Indian Vertisol

**DOI:** 10.1371/journal.pone.0286223

**Published:** 2023-05-31

**Authors:** M. L. Dotaniya, M. C. Nagar, Amita Sharma, C. K. Dotaniya, Rajendiran S., V. B. Singh, R. K. Doutaniya, J. K. Saha

**Affiliations:** 1 ICAR-Indian Institute of Soil Science, Bhopal, India; 2 ICAR-Directorate of Rapeseed-Mustard Research Sewar, Bharatpur, India; 3 Department of Environmental Sciences, College of Agriculture, Gwalior, India; 4 Department of Soil Science & Agricultural Chemistry, Swami Keshwanand Rajasthan Agricultural University, Bikaner, India; 5 Division of Natural Resources, ICAR-Indian Institute of Horticultural Research, Bangalore, India; 6 Department of Agricultural Statistics,College of Agriculture, Gwalior, India; 7 Department of Agronomy, SKN College of Agriculture, Jobner, India; ICAR-Indian Institute of Soil Science, INDIA

## Abstract

Soil fertility management and crop productivity both are inter-related need extensive attention for sustainability. Industries are being built, which over time produces a lot of effluents containing heavy metal(s), which is then dumped on healthy soils and water bodies. Long-term discharge of lead (Pb)-containing wastewater resulted in significant Pb buildup as well as a decrease in soil biological activity. In this experiment, graded dose of Pb, *i*.*e*. 0, 100, 150 and 300 mg/kg and pressmud (PM) (0, 2.5, 5, 10 g/kg) were applied to monitor the Pb toxic effect on soil acid and alkaline phosphatase, dehydrogenase activity. Different treatment combinations were formulated and the experiment was conducted in a completely randomized design (CRD) with three replications. In this experiment, spinach crop was used as a test crop. According to the findings, increased Pb levels in the soil lowered dehydrogenase activity (DHA), acid and alkaline phosphatase. The addition of PM enhanced enzymatic activities by decreasing the labile fraction of Pb in the soil. Incorporation of PM improved the soil enzymatic activities as alkaline phosphatase activity > DHA > acid phosphatase activity in the study. This study suggested that the addition of 10 g/kg PM reduced Pb toxicity (contamination level 300 mg/kg) and improved the soil microbial properties in black soil. These findings are very useful for the remediation of Pb contaminated soil with the help of PM, particularly in peri-urban Pb effluent irrigated areas.

## Introduction

Increasing crop production is essential for feeding the world’s growing population, and soil health play a key role in this. Due to rising population pressure on limited natural resources, the majority of emerging nations are experiencing water scarcity. By 2050, India will need 333 million tonnes (Mt) of food grain up from 289 Mt presently [1]. This is a challenging task for academician and policymakers; since, it focuses on the utilization of organic and inorganic soil amendments for long-term agricultural output [2]. Organic substrate like farm yard manure (FYM) and a balanced application of plant nutrients are being used to improve plant nutrient concentration and soil microbial biomass carbon in low-fertility locations across the globe. Marginal natural resources should be treated scientifically with the required chemical and biological methods before being turned into food crops [3].

Water is one of the natural resources that is fast depleting for agricultural crop production system [4]. Farmers irrigate their crops with low-quality water, which delivers considerable amounts of organic carbon and plant nutrients in the field [5]. Chromium (Cr), cadmium (Cd), nickel (Ni), lead (Pb), arsenic (As), and mercury (Hg) have all been measured in the soil. Excessive amount of these metals in soil adversely affected the crop growth and soil fertility functions [6, 7]. However, it reduced the population and diversity of microorganisms in the soil. Heavy metal(s) effects on microorganisms and soil enzyme activity are also determined and found that increasing trace metal levels have been reduced the enzymatic activities [1]. Contamination of lead in soil might be due to use of Pb containing industrial waste, metal industry byproduct waste and also from Pb containing natural ores [8]. According to the findings, Pb has a negative impact on soil enzymatic activity [9]. Chemical mechanisms, such as metal precipitation as phosphates, carbonates and sulphides; account for the majority of microbial physiological adaptations to heavy metals [10]. Toxic amount of Pb reached in human body via food chain contamination adversely affect metabolic functions of body specially related to nervous and brain system [11]. Physical exclusion, *i*.*e*. exopolymers, and intra-cellular sequestration with low molecular weight cysteine-rich cyst are the important mechanism of Pb exclusion by plant [12–14].

Sugarcane factories role in economic development are well known; on the other hand, produce a lot of pressmud (PM) round of the year in most of the Indian states. In India, sugarcane mills producing more than 8 million tonnes of pressmud each year. Pressmud is a rejected sugarcane waste that, when accumulated, causes storage issues as well as pollution in the surrounding areas of the sugar mill [15]. It is a soft, spongy, amorphous, dark brown to brownish material formed as a byproduct of the sugarcane industry during the carbonation or sulphitation of sugar [16, 17]. Madhya Pradesh state is having three large scale sugar industries at Indore, producing significant amount of pressmud. However, adjoining state like Uttar Pradesh and Maharashtra having larger number of sugar industries and also producing huge volume of PM [18]. When 100 tonnes of sugarcane are crushed, approximately 3 tonnes of pressmud is created as a byproduct [19]. Its sugar content aided in the decomposition of organic matter in the soil and positively improved the soil properties [3, 20]. Pressmud is a good source of organic carbon and may be utilized as a fertilizer as well as a soil amendment [21]. The organic matter and sugar content are eaten by soil biota as food [22]. The mineralization of pressmud produces a range of organic acids during decomposition and ions that form complex bonds to adsorb toxic metal ions [17, 23]. As a result, PM could be used to treat soils that have been contaminated with lead. Soil enzymes are mediated the soil organic matter (SOM) decomposition and plant nutrient kinetics in soil. In this experiment spinach crop was used as a test crop, due to leafy nature and accumulation of more metal contamination and directly affect the food chain content in human life [10]. In light of this, a hypothesis was developed to determine the impact of PM on soil acid and alkaline phosphatase, dehydrogenase activity in Pb contaminated soils under spinach cultivated pots. During the course of the investigation, soil enzymes were measured at graded levels of Pb and PM were applied to Vertisol under spinach crop.

## Material and methods

### Experimental details

The Division of Environmental Soil Science, ICAR-Indian Institute of Soil Science, Bhopal (Madhya Pradesh) undertook a pot culture study. It is located at 26° 16” N latitude and 77° 36” E longitude, with semi-arid and sub-tropical dry summers and freezing winters.

### Collection and analysis of soil

An auger was used to collect bulk soil sample from the research farm to a depth of 15 cm. The collected soil was air-dried, crushed with a pestle and mortar, and then sieved with 2 mm sieve. Singh et al. [24] elaborated analytical procedure(s) was used to identify the physico-chemical parameters of the experimental soil. The soil texture was measured using Bouyoucos’ hydrometer method [25]; it was clay loam in texture and classified as Vertisol, a Hypothermic family of Typic Haplusterts often known as “black cotton soil.” The pH of the soil was 7.81, and the EC was 0.53 dS/m. The chemical composition of the experimental soil revealed that it has a lower range of organic carbon (0.44%) and potassium (216 kg/ha), as well as a low range of available nitrogen (142 kg/ha) and phosphorus (8.64 kg/ha). In terms of available micronutrients, Zn (0.88 mg/kg) was in the deficient class; whereas, Fe (6.15 mg/kg), Cu (2.05 mg/kg) and Mn (5.64 mg/kg) were in the medium category. The total Pb content was 46.94 mg/kg, while the diethylene triamine pentaacetic acid (DTPA) extracted value was 1.23 mg/kg.

### Treatment details

In this experiment, Pb levels were applied at 0, 100, 150, and 300 mg/kg soil. The 5 kg treated soils were placed in 48 pots and replicated three times. The QA/QC standard was followed by a cross-examination of the prepared concentration of working solution using inductively coupled plasma-optical emission spectrometry (ICP-OES) (Model ICP-OES; Perkin Elmer Precisely Optima 2100 DV with 0.001 mg/ kg detecting limits; flow rate was 3 ml/ min). The concentration of metals detected (for Pb 100 ± 0.02, 149.7 ± 00.3, and 299.8 ± 0.5) was the same as the concentration of working solution. The pressmud collected, analysed and were applied at 0, 2.5, 5.0, and 10.0 g/kg, by making 16 treatment combinations:

### Analysis of pressmud

Before being used in the experiment, PM was collected from a sugar processing mill near Kanpur and analyzed for its chemical composition. The usual procedures were used to analyze the majority of the soil fertility parameters [26]. Analyzed data showed pH of the PM was 8.63, and the electrical conductivity was 6.82 dS/m. Organic carbon was 16.21%; total nitrogen, available P, available K, and available S were 1.06, 0.13, 0.39, and 0.43%, respectively. Fe, Zn, Cu, and Mn micronutrient concentrations were 2900, 158, 52.3, and 196 mg/kg, respectively.

### Crop cultivation

Spinach variety “All Green” was used as a test crop in this experiment. A total of 25–30 healthy soaked seeds were sown at optimum field capacity moisture. Seeds were planted at equal distances at a depth of less than 1 cm and covered with soil to avoid harm. In each pot, seedlings were kept to the proper plant density (5 plants per pot). The pots were regularly irrigated with deionized water as needed.

### Fertilizer application

The crop received the required amounts of nitrogen, phosphorus, and potassium: 75, 40, and 40 kg/ha, respectively. In the spinach crop, urea, di-ammonium phosphate (DAP), and muriate of potash (MOP) was used as source of fertilizers. In this experiment, N fertilizer was applied in two doses. Half dose of urea and a full dose of DAP and MOP were applied to spinach at planting as a basal dose. The remaining nitrogen dose was applied at 15 and 30 days after sowing (DAS). Recommended crop management practices were used to grow the spinach.

### Soil enzymatic activity analysis

Soil samples were also collected from rhizosphere after crop harvest, processed (passed through 2 mm sieve), and analyzed for soil enzymatic activity. Dehydrogenase activity (DHA) was measured using Casida et al. [27] procedures, which involved mixing 4 g soil with one mL of 3% triphenyltetrazolium chloride and incubation for 24 hours at 37°C. The intensity of the red colour was measured in a spectrophotometer at 485 nm after methanol was added and centrifuged. The Tabatabai method [28] for measuring acid and alkaline phosphatases was used in the experiment. One gramme soil, toluene, modified universal buffer (MuB) with pH 6.5 used for acid phosphatases; whereas, 1 mL p-nitrophenyl phosphate were used to make alkaline phosphatases. After 1 hour of exposure, the absorbance at 440 nm was measured using a Spectrophotometer.

### Statistical analysis

To analyze statistical data, three replications of a Completely Randomized Design (CRD) with a factorial treatment structure were used. The significance of main effects and interaction effects was determined using an analysis of variance. The comparisons of treatments (main effects and interaction effects) were done at a critical deference level of significance (p < 0.05). SAS version 9.3 was used for the entire statistical data analysis [29].

## Results

### Effect of Pb and PM levels on DHA

During the experiment, increasing the Pb levels from 0 to 300 mg/kg drastically lowered the DHA activity. The detected values of DHA (TPF/g soil/24 h) in the treatments 0, 100, 150, and 300 mg/kg Pb were 41.99, 32.91, 29.19, and 20.26 μg TPF/g soil/24 h, respectively (Table 1). PM application, on the other hand, boosted DHA levels from 25.23 to 38.24 μg TPF/g soil/24 h in the control group to 10 g/kg in the PM treatment pots, respectively. Lower PM treatment didn’t significantly (p < 0.05) increase DHA levels, whereas higher PM application i.e., doses 5 and 10 g/kg soil increased DHA levels by 35.87 and 51.56% over control, respectively. DHA activity in soil is influenced by the interaction of PM and Pb levels; the highest concentration was found in the highest PM applied pot, while the lowest concentration was found in the lowest PM applied pot.

**Table 1:** Effect of Pb & PM levels on DHA activity (μg TPF/g soil/24 h).

Pb (mg/kg)	Pressmud (g/kg)				
	0	2.5	5	10	Mean
0	33.60^e^	38.10^cd^	42.35^b^	53.90^a^	41.99
100	27.85^f^	28.11^f^	35.65^de^	40.05^bc^	32.91
150	23.25^gh^	23.40^gh^	37.70^cd^	32.40^e^	29.19
300	16.20^i^	16.85^i^	21.40^h^	26.60^fg^	20.26
Mean	25.23	26.61	34.28	38.24	

### Effect of Pb and PM on acid and alkaline phosphatage activity

Increased Pb levels increased the stress on the spinach crop and decreased the microbial community in the soil. The results of the experiment were used to calculate the impact of Pb toxicity on acid phosphatase activity (Table 2). Increased Pb dose upto 300 mg/kg reduced acid phosphatase activity from 39.06 to 32.93 μg PNP/g soil/24 h; the 15.69% reduction compared to the control treatment. Application of PM, on the other hand, reduced Pb contamination and increased acid release (28.63, 30.80, 32.53, and 34.31 μg PNP/g soil/24 h in control, 2.5, 5, and 10 g/kg applied PM, respectively). During the investigation, the interaction effect of Pb and PM was also calculated. The maximum level of acid phosphatase activity was seen with the highest amount of PM and the lowest level of Pb applied treatments. Alkaline phosphatase activity was assessed during the experiment to establish the Pb toxicity effect and PM mediation potential in soil. The amount of lead in soil had a substantial impact on alkaline phosphatase activity, which decreased from 71.23 to 42.89 μg PNP/g soil/24 h. (Table 3). The application of the highest level of Pb (300 mg/kg) resulted in the largest reduction (39.78%) in alkaline phosphatase activity in comparison to control. When the PM application rate was increased from control to highest level (10 g/kg soil), the alkaline acid activity rose from 34.99 to 75.66 μg PNP/g soil/24 h. The both acid and alkaline phosphatase activity improved with the highest level of PM and the lowest level of Pb. After the harvested of spinach crop, soil samples were taken from each pot and analyzed for soil pH, EC (dS/m) were ranged 7.77–7.79 and 0.48–0.51, respectively. Soil organic carbon mean value in the treatment 0, 2.5 and 5 were 0.45%, 0.49 and 0.51%, respectively. We also measured the Pb content in soil and found 88.15, 129.31 and 280.32 mg/kg; whereas, extractable concentration was 1.42, 1.53 and 1.58, in 100, 150 and 300 mg/kg applied Pb, respectively.

**Table 2:** Effect of Pb & PM levels on acid phosphatase activity (μg PNP/g soil/24 h).

Pb (mg/kg)	Pressmud (g/kg)				
	0	2.5	5	10	Mean
0	30.01^gh^	33.00^d^	35.94^b^	39.06^a^	34.50
100	29.52^hi^	31.98^e^	33.18^d^	34.28^c^	32.24
150	29.03^i^	30.62^fg^	31.01^f^	30.93^f^	30.40
300	25.96^k^	27.58^j^	29.99^gh^	32.93^d^	29.11
Mean	28.63	30.79	32.53	34.30	

**Table 3:** Effect of Pb & PM levels on alkaline phosphatase activity (μg PNP/g soil/24 h).

Pb (mg/kg)	Pressmud (g/kg)				
	0	2.5	5	10	Mean
0	42.72^f^	57.08^d^	70.40^c^	114.72^a^	71.23
100	37.20^g^	51.84^e^	67.56^c^	77.32^b^	58.48
150	32.68 ^g^	50.20^e^	61.60^d^	66.60^c^	52.77
300	27.36^h^	49.76^e^	50.44^e^	44.00^f^	42.89
Mean	34.99	52.22	62.50	75.66	

## Discussion

Among the enzymatic activities, DHA was drastically affected by the Pb toxicity. As a result, it may be a helpful biomarker of Pb toxicity for soil health protection. Many researchers have observed similar findings [30–32]. DHA may be a good indication of microbiological activity in the soil since it reflects the full range of oxidative activity of soil microflora. In the early phases of soil organic matter oxidation, dehydrogenase activity transports hydrogen and electrons from substrates to acceptors [33]. Welp [34] found that heavy metals, particularly Pb, decreased soil dehydrogenase activity by 10–90% depending on the rate and kind of metal used. Heavy metals prevalent in soil, such as Cu, Zn, and Pb, make DHA vulnerable. DHA levels in agricultural fields were reported low (4.4 g TPF/g dw/h) due to the presence of lead [32]. In many agricultural crop trials soil health parameters were improved when PM was added as a potential amendment [15]. Because of its high sugar content, it accelerated the decomposition of organic matter in soil [3, 20]. Pressmud is a good source of organic manure that may be utilized to improve soil fertility and crop productivity [17, 21, 22]. It requires more storage space in addition to proper disposal. The organic components and sugar content are used as food by the soil biota. According to Klik et al. [35] reported that increasing organic matter deposition by sewage irrigation boosted DHA levels while reducing Pb toxicity.

Microbial activity is influenced by the Pb levels in the soil. In general, there is a negative relationship between Pb content and microbial activity. Pb toxicity in soil lowered the activities of dehydrogenase, urease, catalase, and acid phosphatase by 5.3–74.8% when compared to the control [36]. Pb levels were employed in the soil to reduce enzymatic activity as well as microbial population and diversity [14, 37–40]. It reduced the soil enzymatic activity and the amount of food available to soil bacteria by preventing the mineralization of organic soil components [41, 42]. Plant nutrient mineralization rate and soil properties are adversely affected by the metal concentrations. All of these factors are worked together to slow down the release of plant nutrients from organic matter into the soil solution [43]. The suppression of soil enzyme activity by Pb metals is a complex phenomenon involving several variables. Lead in soil induces changes in microflora composition and enzyme activity on a quantitative and qualitative basis [44]. Bangar et al. [23] reported that addition of pressmud improved the soil health by improving the concentration of NPK and microbial parameters. These soil parameters boost up the soil microbial diversity and improved the sugarcane yield. The inclusion of organic compounds led in the production of a variety of organic acids and complexes that mediated soil Pb poisoning [45]. When an addition of organic matter, the pH of the soil was altered; this is allowed the soluble portion of Pb to form a complexation or bond with ions and immobilized it in the soil [46]. Increased bio-available percentages of heavy metals, according to these researches, can reduce soil enzyme activity by associating metal ions with the sulfhydryl group of the enzyme or chelating with the enzyme substrate. When organic or inorganic additions are used in soil for Pb metal cleanup, microbial activity may vary [47]. Addition of pressmud in soil enhanced the source of C and elevated the population and diversity of soil microorganisms. It mediated the SOM mineralization kinetics and free the plant nutrient from complex molecule to inorganic ions [48]. Secretion of low molecular organic acids during decomposition, modify the humus metal complex and reduced the lead toxicity in soil [49]. Pressmud is a rich source of plant nutrients because it has a good impact on the physical, chemical, and biological characteristics of soil [50].

## Conclusions

Heavy metal(s) dynamics in soil are mediated by organic and inorganic compounds, which enhance soil health parameters. The majority of lead-contaminated agricultural soils have issues with soil organic carbon mineralization because of low levels of soil microbial population and diversity. In this study, pressmud was applied to the soil to enhance soil organic carbon, which in turn reduced Pb toxicity and increased the microbial community. Adding 5 g/kg pressmud at the highest contamination level (300 mg Pb/kg) reduced Pb levels and improved soil enzymatic activities, including DHA (52%), acid phosphatase activity (20%), and alkaline phosphatase activity (116%), when compared to control. The enzymatic activities of alkaline phosphatase > DHA activity > acid phosphatase were improved by adding PM to the soil in this study. In summary, according to this study, applying 10 t/ha PM into the black soils reduced the extractable Pb concentration (at contamination level 300 mg/kg) and enhanced soil microbial properties and enzymatic activities.
